# Sources of Information on HIV and Sexual and Reproductive Health for Couples Living with HIV in Rural Southern Malawi

**DOI:** 10.1155/2013/235902

**Published:** 2013-04-15

**Authors:** Belinda Chimphamba Gombachika, Ellen Chirwa, Address Malata, Alfred Maluwa

**Affiliations:** ^1^Department of Community Medicine, Institute of Health and Society, Faculty of Medicine, University of Oslo, P.O. Box 1130, Blindern, 0318 Oslo, Norway; ^2^Faculty of Nursing, Kamuzu College of Nursing, University of Malawi, Blantyre Campus, P.O. Box 415, Blantyre, Malawi; ^3^Faculty of Nursing, Kamuzu College of Nursing, University of Malawi, Lilongwe Campus, Private Bag 1, Lilongwe, Malawi

## Abstract

With wider access to antiretroviral therapy, people living with HIV are reconsidering their reproductive decisions: remarrying and having children. The purpose of the paper is to explore sources of information for reproductive decision used by couples living with HIV in patrilineal and matrilineal districts of Malawi. Data were collected from forty couples from July to December 2010. Our results illuminate five specific issues: some of the informants (1) remarry after divorce/death of a spouse, (2) establish new marriage relationship with spouses living with HIV, and (3) have children hence the need for information to base their decisions. There are (4) shared and interactive couple decisions, and (5) informal networks of people living with HIV are the main sources of information. In addition, in matrilineal community, cultural practices about remarriage set up structures that constrained information availability unlike in patrilineal community where information on sexual and reproductive health, HIV, and AIDS was disseminated during remarriage counselling. However, both sources are not able to provide comprehensive information due to complexity and lack of up to date information. Therefore, health workers should, offer people living with HIV comprehensive information that takes into consideration the cultural specificity of groups, and empower already existing and accepted local structures with sexual and reproductive health, HIV, and AIDS knowledge.

## 1. Introduction

When confronted with potentially life-threatening illness such as cancer, HIV, and AIDS, information may provide needed knowledge about the disease, treatment, and self-care management. It may also facilitate coping by mediating uncertainty and anxiety by providing social support [1, 2]. This paper therefore explores sources of information on sexual and reproductive health, HIV, and AIDS that concordant couples living with HIV (CLWH) in Malawi use in making reproductive decisions.

HIV and AIDS information is an important resource for people living with HIV (PLWH) [3, 4] and remains the most important tool in HIV and AIDS management [5]. In the early days of the epidemic, information about HIV and AIDS was critical resource to prevent transmission of HIV and manage the complications that accompany HIV and AIDS [6]. Huber and Cruz [7] allude that where HIV and AIDS are concerned, a large portion of the affected population has been and continued to be active in the pursuit of relevant information in order to be able to make informed decisions. However, HIV and AIDS information is complex, multidisciplinary, and voluminous; in addition, much of the HIV information is published and distributed outside the traditional/local channels, for example, orature, traditional ceremonies that are therefore not part of standard clinical information resources. Access to HIV and AIDS information is difficult given its limited dissemination and integration into the usual streams of health information [5, 8].

In Malawi, HIV poses major challenges for public health. At a prevalence rate 10.6%, it has one of the highest national HIV prevalence rate in the world [9]. Heterosexual contact is the principal mode of transmission and about 88% of all new HIV infections in Malawi are acquired through unprotected heterosexual intercourse [10], while mother-to-child transmission (MTCT) accounts for 25% of new HIV infections. Antiretroviral therapy (ART) has played an important role in decreasing perinatal HIV transmission to less than 2%, thereby reducing the women's concern regarding HIV transmission to their infants in Malawi [11]. In the early years of the HIV epidemic, little attention was given to the reproductive decisions among people living with HIV (PLWH) because of the risk of mortality and few options to reduce mother-to-child transmission [12]. In recent years, HIV-related morbidity, mortality, and mother-to-child transmission of HIV during pregnancy, delivery, and in the newborn are declining because of ART [13].

Evidence emerging from research in developing countries indicates that ART may encourage PLWH receiving treatment to reconsider their reproductive decisions including getting married and having children. These decisions are related to perceptions that ART allows them to live longer, and have more “normal” social sexual lives and possibly have healthy, HIV-negative children [13–18].

Despite the growing interest regarding provision of treatment and prevention of mother-to-child transmission of HIV (PMTCT), little is known about the extent to which these CLWH get their information about sexual and reproductive health. Gruskin et al. [6] indicate that decisions about child bearing are complicated because apart from the CLWH considering the health of the woman and the risks of vertical transmission, in some countries, they face policies that reduce their reproductive freedoms. Several studies from South Africa, Brazil, USA, and Europe have shown hindrances for PLWH in accessing information pertaining to sexual and reproductive health from the health workers because it was considered socially unacceptable for PLWH who had publicly disclosed their status to have more children [19–21]. However, changes in our health care system have put more responsibility on patients to be active participants in decision-making, self-care, and disease management. Active participation presumes that the person will have the necessary information [2].

So far, studies about information and PLWH have been done mostly in developing countries using quantitative research methods. These have mainly focused on the internet as a source of information and PLWH information needs in order to boost their health and well-being [2, 7, 22]. Recognizing these gaps in sexual and reproductive health in Malawi, a descriptive qualitative study was conducted in two rural communities in the southern part of Malawi. The study was part of a big study that was looking at reproductive decisions of CLWH, which was conducted in matrilineal and patrilineal communities: for comparison purposes due to its cultural differences, particularly in terms of kinship organization. In patrilineal Chikhwawa women leave their natal household to live in their husbands' compound after marriage (virilocal). Marriage was legitimized by transfer of cattle, but nowadays bride price in monetary equivalent, *lobola,* is paid to the bride's family. Traditionally, when the husband dies, the woman may remain in her husband's homestead where she together with her children will be taken care of by the relatives of the late husband [23]. In case of economic constraints, the assistance provided might be inadequate. In some situations, wife/widow inheritance is opted for where the late husband's young brother or cousin inherits the widow and provides for her and her children's needs. He could also have children with her. If the widow is not inherited, she goes back to her home village [24]. By contrast, in matrilineal Chiradzulu, marriage is followed by uxorilocal (matrilocal) residence where men leave their natal household to live in those of their wives after marriage. In cases of divorce or death, children often maintain relations with their fathers throughout their life even though they may live with several of their mother's successive husbands [23]. These cultural practices enhance the transmission of HIV/AIDS. Through the analysis of interactions in the two contexts, it allows us to begin to understand the broader aspects and influence of contextual factors on information seeking for reproductive decisions by CLWH [24].

## 2. Materials and Methods

A qualitative approach was deemed most appropriate because there is very little existing research that has been conducted thus far. In addition, the exploration of the sources of information for reproductive decisions in CLWH involves sensitive, emotive, and personal topics that can be best captured through careful probing using the qualitative in depth interview (IDI).

### 2.1. Study Setting

The informants were recruited from ART Clinics involved in the treatment and care of PLWH, which were Ngabu Rural Hospital in Chikhwawa and Ndunde Health Centre in Chiradzulu districts. These sites were chosen because they received patients from remote villages, away from trading centres and main roads that attract people that do not originate from the study districts which was the aim of the main study. The two centres offer inpatient and outpatient HIV and AIDS treatment using multidisciplinary teams and serve primarily low-income individuals from diverse backgrounds. Ndunde is located 7 km from Chiradzulu district's headquarters while Ngabu is 60 km from Chikhwawa district's headquarters. The catchment areas of these two study sites face challenges ranging from food insecurity, low accessibility to safe water, low household income levels, and poor communication infrastructure coupled with high prevalence of HIV and AIDS. The communities have access to earth roads, though during the rainy season, the conditions of the roads deteriorate and communication becomes a problem. The informants came from catchment areas surrounding these health facilities, and the average time from their villages to the hospital ranged from 1 to 3 hours on foot [25].

### 2.2. Data Collection

Informants were recruited upon receipt of permission to conduct the study following ethical approval from Ethical Review Boards in Malawi and Norway. Twenty CLWH were recruited in the study using purposive sampling. Informants that were HIV positive (concordant couples), had informed about each other's HIV status as a couple, and were in the reproductive age group of 18 to 49 years [25] were recruited for the study. Informants were sampled to saturation, and data collection stopped when there was no new information being gathered from the interviews.

Data, which was part of a larger study on reproductive decisions of CLWH in southern Malawi, were collected from July to December 2010. A total of three months were spent in each study setting in order for the researcher to internalize, rather than observe patterns of beliefs, fears, expectations, dominant ideas, values, and behaviours of the informants. The researcher gained access to the ART clinics through collaboration with the ART clinic coordinators. In both sites, the clinic coordinators identified a contact person who oriented the researcher on the clinics' objectives and strategies.

The potential informants were approached while waiting for their monthly check up at the two study sites. The information about the research was given to the ART clinics in two phases. Firstly as part of the general briefing (Health talk) that all clients received in an open area before the check-up begins and secondly as a private conversation in a private room with the contact person. Those who wished for further information about the study or were interested in participating in the study informed the contact person. Couples living with HIV, who indicated willingness to take part in the study, were asked some questions to determine eligibility. Those eligible were accorded an appointment for an interview. Informants gave an oral consent, which was tape-recorded. The informants that consented were assured of confidentiality. The researcher informed the informants that they were free to withdraw from the study if they wished. During the research activity, all the informants were given transport reimbursements of $2 and were provided with snacks.

The IDIs were conducted in vernacular language. Indepth interviews were opted for because they allowed room to explore issues deeper are interactive in nature thereby enabling clarification of issues during the interview. In addition, they allowed further probing and modification of interview guides in the course of the study [26]. The couples were interviewed independently from their partners but on the same day to enable free expression of feelings and views. The interviews lasted between 50 minutes to 2:30 hours at a private place determined by the informants where confidentiality was guaranteed.

### 2.3. Data Analysis

The general principles and procedures for qualitative data content analysis as summarized by Weber, [27] and Krippendoff, [28] were used. All data was recorded with an audio recorder, then transcribed verbatim. The transcribed data were translated back into English. The content of the interview text was from a larger study that was exploring reproductive decisions of CLWH. The interviews were read several times to obtain a sense of the whole. Then the texts about sources of information were extracted. During the initial phase of the analysis, a coding team of three coders created a series of predefined preliminary codes from phrases of similar meaning that emerged in some of the transcripts. The coding team then independently analysed each transcript and assigned the predefined codes that were embedded in the interview guide: media, peers, kin and health workers. In order to ensure interrater consistency, tentative categories of the codes were discussed between three researchers who initially did the coding independently. Once the codes were agreed upon, the underlying meaning of the different categories of the codes was formulated into themes. In the following section, additional details regarding the themes have been provided in form of quotations. NVivo 9 qualitative data analysis software was used to assist in organizing data.

The findings and discussion are reported under the four predefined codes: media, peers, kin, and health workers.

## 3. Results

### 3.1. Characteristics of Informants

The informants for the study comprised twenty couples (forty informants) whose age ranged from 23 to 49 years old with a mean age of 38 years. Twenty-six informants (ten from the matrilineal and 16 from the patrilineal communities) had been living with HIV for less than five years. All couples reported that they were in a monogamous relationship. They were living in either the wives or husbands' natal compounds depending on kinship organisation. Six informants had no formal education at all. Thirty-four informants had some schooling; 28 (14 from each community) had completed primary education, while the other six had completed secondary school education (Form 4). Thirty-three of the informants were subsistence farmers with small gardens and were without any other source of income. Only seven male informants had formal jobs. All the informants were Christians.

Table 1 gives an overview of marriage and children of the informants. Following divorce or death of a spouse, the informants remarried. Some established new marriage relationships with spouses living with HIV and they continued to have children in their new marriages alongside having children from the previous marriages. It was prior to making decisions about these experiences that the informants sought information.

### 3.2. Sources of Information

The purpose of the study was to explore sources of sexual and reproductive information among CLWH in matrilineal and patrilineal communities. There was a wide range of sources of information that informants sought when making a decision. This information was, however, comprehensively sourced from radio, peers, and kin and not directly from the health workers. The only difference for the two communities in the sources of information was the use of kin.

In the patrilineal society, issues of sexual and reproductive health and HIV-positive status are discussed during their traditional marriage ceremonies whether it is a first or subsequent marriage, contrary to the matrilineal society in which such information is given only during the first marriage. All informants indicated peers as the main source of information for marriage and childbearing decisions, while kin was mostly used for marriage decisions only. Use of the media, radio, and print was indicated by 36 and 28 of the informants, respectively. Only two women sourced information on child bearing from health workers.

As the informants narrated their experiences from this question: *Can you tell me how you go about seeking sexual and reproductive health information?* They elaborated how they initiated it, how they explored the different sources of information, and how they used the information at the end of the search. The informants were very proactive in searching for the information. They indicated that they sought the information on their own which some informants referred to as “*phukusi lamoyo wako samakusungira munthu wina” *literally translated as *“You are the custodian of your own life*.” As indicated by one informant from the matrilineal society: “*This is my life, I am the custodian of my life and therefore I have to go out there myself for the information.*”

One couple in their separate interviews eloquently captured this by stating

*“Although we discussed with my wife about my intentions to have a child, we needed more information about it. We decided to search for information. The hospital was out because their message is clear-cut HIV-positive no children so I decided to bring the issue to my friends living with HIV at the ART clinic. They are the ones who referred me to…. After a week, I contacted him. He was a group leader of a support group and acted as a local counsellor for people living with HIV. He told me that we had to join his support group where such issues are shared. I discussed with my wife who accepted to join the support group. It was at the support group that we got a lot of information about preventing transmission of the virus to the child.” Man from matrilineal community.*

*“Since my husband had been demanding for a child, I though it wise to search for information from experienced women. The first woman I approached at the clinic [ART] told me to ask the nurses. I did not even dare to ask them [nurses] since we all knew that we were not supposed to have children. Another woman told me that she never attended antenatal care for fear of being reprimanded by the nurses. She only presented herself at the hospital (health centre) when she was in labour. When she was asked to produce her antenatal card, she lied that she had forgotten it and only showed her ARV cards. She never gave me comprehensive information. Fortunately, my husband told me that his friend had advised him that we must join a support group. It was at the support group meetings that we got information on what to do when I am pregnant and breast-feeding. The other practical things like administration of Neverapine [Sic] to the baby and issues of child follow up I was told at the hospital after I delivered my baby.” Woman from a matrilineal community.*

They all indicated that at this stage they were anxious as reflected by a woman from the patrilineal community:

*“We were afraid that my husband's marriage counsellor would shout at us because of our decision to have a child but we were surprised with his approach to us, he was very understanding. He told us politely that he did not know what the hospital says on these issues so he referred us to another man who was also living with HIV for further information.”*

While a man from the matrilineal community in his second marriage who wanted information about remarriage following divorce indicated

*“Although I was a member of the support group, I was not sure where to start from, I was not sure if my friends would assist me.”*

The informants would subsequently move on to other sources until their needs were fulfilled. All through, nearly all the informants would be reviewing and discussing amongst themselves if they had adequate and reliable information. It was during this reviewing and discussion that they discovered a need for expert information which according to their narrative only two women managed to visit the health workers at the hospital.

### 3.3. Information from the Media

The informants depend on the media for any other significant information and news pertaining to sexual and reproductive health, HIV, and AIDS. They indicated that there is a lot of information on the radio about reproductive decisions for PLWH. One of such programmes was the “*Mungathe*”, that is, “*It is possible*”. This programme advocates that it is possible for PLWH to have HIV-negative children provided they follow stipulated guidelines. However, there was at times conflicting information between the media and the hospital as narrated by one informant:

*“There is an advert that is aired frequently on the air known as ‘MUNGATHE' ‘We can make it'. The main message is that although a person has HIV, they can still have a child and those who really want to have a child can go and discuss with nurses at the hospital. As such these messages act as a driving force for the couples to get pregnant.” Male from matrilineal community.*

Apart from pictures on condom use, all informants from the two study sites indicated that the health workers never use pictures or posters during counselling or health education on sexual and reproductive health, HIV, and AIDS.

### 3.4. Information from Kin

Traditional fora for communicating important information in the patrilineal society were related to marriage formalities: *Luphato *(when a gift in the form of a piece of cloth “*chitenje*” and money is given to a girl/woman from her boyfriend as a sign of marriage proposal which she has to show to her parents),* Chifunukura mulomo* (when money is given to a girl by her boyfriend in order to initiate discussions of marriage proposal),* Maonano* (when money is given to a girl's parents from the boy's parent to initiate first discussions about marriage issues), and* Lobola* (Bride price). These marriage fora are the most recognised channels for disseminating important messages including issues of HIV-positive status. One female informant from the patrilineal community eloquently captured this theme by stating the following:

*“Then he gave me a scarf and K500.00 which I received happily and showed them to my parents. My mother asked me if I had disclosed to him about my problem (HIV) and I told her that I did. Then his relatives came to give my relatives money for “lobola” and they discussed the issue regarding our status (HIV) and what we must do to live longer.”*

The informants indicated that the information is usually not comprehensive and the elders conclude with the words: *“follow the hospital advice diligently.” *In contrast, the matrilineal informants indicated that such information is never discussed during their subsequent marriage ceremonies.

*“The problem is that they just leave us on our own and they say, “Just leave them, they will know what to do themselves this is not their first marriage.” Male from matrilineal community.*

### 3.5. Information from Peers

Information was also obtained from friends who were also living with HIV. They acknowledged that even though peer and kin shared some similarities especially in offering psychological support, they recognized some critical differences between these two sources. The information was sought from PLWH because they had accumulated wisdom through reflection on their lived experiences. They narrated that the experiential information was also blended with biomedical information. They described the source of information as emotionally rewarding because it also helped them to reduce their feelings of uncertainty and anxiety over their decisions to either remarry or have children while they were living with the HIV. This group's views can be summed up by informants who said

*“In these support groups, we advice each other that we must not be promiscuous because in so doing we are, either, adding more virus in our body or shortening our lives. We also discuss issues related to the medication (ARVs) and that an HIV-positive person has the right to get married.” Male from matrilineal community.*

*“It does not mean that having the virus is the end of one's life, no, there are people who know that that person is on medications and they still approach her for marriage, it is happening. These issues are also discussed at the support group.” Male from patrilineal community*

Others sought information in order to inform their friends or spouses so that they make informed decisions about marriage and child bearing.

### 3.6. Information from Health Workers

All the informants indicated that the hospital had reliable knowledge and expertise in relation to sexual and reproductive health, HIV, and AIDS. However, 12 out of the 20 couples, seven from the matrilineal, and five from the patrilineal communities had children after knowledge of their HIV-positive status. Out of these only two women, one from each study site had consulted their health workers of their intentions to become pregnant. However, these two informants explained that they were shouted at. They regretted having gone to ask.

The majority indicated receiving advice from the health workers against becoming pregnant. Informants also mentioned judgemental tones from the health workers when they could not follow the advice on pregnancy prevention:

*“The doctors do not encourage it because we are told that we must not have children but when we go back home, we just do it as a couple and when we come back pregnant they shout at us.” Female from matrilineal community.*

*“The health personnel shout at us. They say that they give us condoms to use but surprisingly the women come back pregnant. They further ask us if we are really concerned with our health because anyone with HIV is not supposed to get pregnant.” Male from patrilineal community.*

The time spent with the health workers during consultations at either the ART or Maternity clinics, was longer during the first visits. During the consecutive visits, counselling hardly took place. The majority of the informants complained about lack of support, communication, and understanding by the health workers on the value of marriage and child bearing during the consultations. Lack of comprehensiveness in the information they got was a problem as cited by these informants:

*“Information on sexual and reproductive health is given but it is not adequate, it is as if they skate around these issues, we expect that the health workers will tell us everything.” Man from matrilineal community.*

*“They include issues about sexual and reproductive health at the antenatal clinic. Otherwise at the ART clinic that type of information is not included in the health education messages.” Woman from patrilineal community.*

They explained that the form of question was often closed-ended and leading: how are you? Are you facing any problems? were some examples of the questions that the informants gave. Informants related this to the health workers' ever-busy schedule in attending to the long consultation queues at the ART clinic. Consequently, the informants did not ask any questions to the health workers to avoid “taking up too much of the health worker time” when other patients were waiting to be seen. Lack of privacy at the clinics, limited local health facilities, and long distance to the health facility were some of the reasons that the informants gave for opting to get information from friends:

*“They are in a hurry you cannot even ask questions, they just greet us: ‘How are you this morning, do you have any problem today?' So if you have a problem you inform the doctors that such and such is bothering me and they just prescribe the drugs for you.” Woman from matrilineal community.*

Male spouses indicated that although there was a government policy that men must be involved in the antenatal health education classes, the fathers who wanted to be involved in the care often felt excluded except for the HIV blood tests that were mandatory for every pregnant couple.

## 4. Discussion

The narratives have shown that informal networks of people living with HIV are the main sources of information for reproductive health decisions. In addition, they have highlighted five issues. The first is their decision to remarry after divorce or death of a spouse; then the establishment of a new marriage relationship with a spouse living with HIV; and the decision to have children in new marriages despite being parents from previous marriages. These issues created a need for information. Secondly, a shared and interactive couple decision also required information. Thirdly, the results show that the context or situation previously mentioned which were the main drivers to information-seeking were much more powerful predictors of information seeking behaviour than demographic factors [29]. Fourthly, cultural practices about remarriage set up structures that constrain information availability especially in the matrilineal community unlike in patrilineal community where information on sexual and reproductive health, HIV, and AIDS is disseminated during remarriage formalities. Finally, some of the information was either inaccurate or inadequate, indicating the importance of availability and accessibility of reliable information for sexual and reproductive health decision making.

As the informants narrated their experiences, an information-seeking trail emerged. The informants narrated how they initiated it, how they explored the different sources of information, and how they used the information at the end of the search. The process involved first a discussion on whom they must consult for further information and between the two, man and woman, if it was an issue to do with marriage or between husband and wife, if it was an issue to do with childbearing. This interactive process for solving problems has also been documented by Kelman and [30] Mbweza et al. [31].

### 4.1. Peer Sources of Information

The results show that the informants often begin a search for information by consulting other people also noted by Case [29] and Brashers et al. [1]. Peers were easily accessible and the information was accompanied by emotional support. Personal channels are also better suited to handle special individual needs and questions due to its characteristic of being able to provide immediate feedback [32]. For many information seekers, the emotional experience associated with receiving information was as important as the quality of the information itself [33, 34]. Compounding the situation was the long distances from homes to the hospital, with little public transportation. The implication is that CLWH support groups were the readily means of obtaining sexual and reproductive health, HIV, and AIDS information due to their availability as they were closer to their homes. While this may be related to the ease with which one can consult others, certain people are more likely to be chosen as information sources than others [4]. A person is more likely to choose an individual who is in similar situation or has sound knowledge in the issue. Socially similar people may be chosen as information sources partly because of opportunity; people who are similar to one another in terms of race, social class, or geographic proximity are more likely to interact. Moreover, socially similar people are more likely to have comparable life experiences, increasing the odds that they will understand one another's social circumstances and hold similar values [34, 35]. Rogers [36] describes it as homophilous, which is an obvious principle of human communication that the transfer of ideas occurs most frequently between individuals that are similar. Homophily may produce these patterns because individuals who share social statuses tend to hold similar values and are more knowledgeable about one another's circumstances, resulting in greater empathy [35].

However, although peer-based information exchange filled an information gap to these informants, the preferred peers were not able to provide comprehensive information due to both the complexity and their possible lack of up to date information on sexual and reproductive health, HIV, and AIDS issues. We therefore suggest that they must get extra information from formal or more educated information sources, because situations arise when they misinterpret meaning of biomedical issues or overgeneralize situations as they interpret personal stories [37, 38]. Home visits by health workers can be one way of bringing the messages closer to PLWH. The topics of discussions should include superinfection, prevention of unintended pregnancies, protection of the unborn babies from HIV through PMTCT strategies, and how to cope and manage issues related to childbearing. However, this intervention is supposed to begin with the positive aspects of culture and behaviour [39], which in this case are marrying and having children rather than focusing only on the negatives behaviours currently observed in the informants.

With low staffing levels in the rural hospitals that serve the two study settings as highlighted by CLWH, support groups remain vital in provision of sexual and reproductive health, HIV, and AIDS information. These findings highlight a need to further develop information services currently being offered by PLWH in their support groups in order to effectively deliver important messages to the rural communities. First, we suggest mechanisms that are nonjudgemental and open to be put into place at the hospitals where the group leaders would know where and who to contact when the need for further clarity or information arises. Secondly, the public health workers in HIV and AIDS must explore other activities of this channel and learn more about the skills and knowledge that the leaders in these groups have. Finally, the group leaders in these support groups must undergo extensive training in both sexual and reproductive health issues, HIV, and AIDS.

### 4.2. Kin Source of Information

Traditional marriage formalities initiated by village elders served as one of the most recognised channels of spreading information on important matters in the patrilineal but not in the matrilineal societies. In some African communities, people live largely through their forms of indigenous communication with the most important site being the village [39, 40]. In patrilineal Chikhwawa, all the formalities of marriage are strictly followed during remarriages probably due to the bride price that is still being paid. It is during these marriage formalities that issues pertaining to sexual and reproductive health, HIV, and AIDS are discussed. Unlike in matrilineal Chiradzulu only the first marriage deserves formal traditions including counselling. This is because the first marriage represents a process of becoming an adult and a major transition in the establishment of an independent household with long-term commitments to another person. The lack of counselling in subsequent marriages in matrilineal Chiradzulu is a missed opportunity to communicating reproductive health, HIV, and AIDS issues to these couples that are living with HIV.

In sub-Saharan Africa, older adults play a crucial role as educators and care givers and they remain influential community members and leaders. They also act as gatekeepers of information, playing a major role in reinforcing attitudes and normative behaviour [41]. A South African study showed that providing HIV education workshops to older people led them to a more positive attitude towards people living with HIV and to perceive themselves as more able to provide information and care to PLWH [42]. However, despite this fact, rarely do health education efforts involve the elderly people in a society. Hospital personnel usually have disparaging attitudes towards the role that the elders can do and do play in their communities [43]. Therefore, in the rural societies of this study setting, using elders and oral tradition forum for transmitting knowledge on sexual and reproductive health, HIV, and AIDS as information carriers will provide a favourable platform because it utilizes already existing and accepted structures [44, 45]. However, the promotion and adoption of this approach in the patrilineal and matrilineal traditional settings require that local counsellors, who in this case are the already existing village elders, to be well trained by experts so that they are conversant with sexual and reproductive health, HIV, and AIDS issues.

### 4.3. Mass Media Source of Information

The results that the radio was one of the main sources of information on sexual and reproductive health, HIV, and AIDS have also been reported by the NSO, [25] although being used as a source of general information. In Malawi radio is affordable by most households due to their low running costs. Ever since the government of Malawi liberalized the media industry, there has been significant growth in the number of Frequency Modulation (FM) radio stations across the country. In these two rural communities, more than two FM radio stations, Zodiac, MBC 1, and 2 are received clearly. Radios are convenient to use, reach large audiences at the same time, and programmes that are in local languages have an impact on the community [5, 32]. Although they have been criticized of having less credible information and may downplay risks associated with unhealthy practices [46]. However, there were clashes between the information from the media and the health workers in terms of PLWH and childbearing since they were not operating from one authority. Just as in any communication process, some overlaps and gaps are bound to exist especially where multiple agencies are involved in similar programmes [47]. In such cases, coordination between the main health service delivery system, Malawi Ministry of Health, and the other formal and informal communication networks before messages are produced is essential.

Print material was not a main source of information for the informants. In Malawi, a lot of information that has been published and distributed is not readily accessible to the rural masses owing to high illiteracy rate [25]. Illiteracy presents a challenge to using written media as a channel of communicating sexual and reproductive health, HIV, and AIDS issues. This is compounded by the low intensity and quality of information, education, and communication materials (IEC) in rural and hard to reach areas where majority of the population live [48]. Furthermore, despite the fact that English is the official language many people in Malawi do not understand it, therefore to those who are able to read, materials printed in English will still be ineffective. Similar patterns were observed in Tanzania, [49], South Africa, [50] and Zimbabwe [51].

These results therefore suggest that there is a need for the materials to be available in the vernacular language. Similarly, radio programmes on sexual and reproductive health, HIV, and AIDS should also be in the vernacular language and be broadcasted at a suitable time for the target population. This is important because mass media are most effective means of communication for changing weakly held attitudes where the adoption of the new behaviour does not represent a large degree of risk or uncertainty. However, issues about sexual and reproductive health, HIV, and AIDS may be a new challenge to the couples, and when the messages challenge strongly their attitudes such as traditional norms of reproduction, there is a high level of uncertainty. In such cases, Rogers, [36] emphasized that the most effective approach to use is the interpersonal channels of communication which informants in the study relied on as indicated in the previous sections.

### 4.4. Health Workers Source of Information

The informants expressed dissatisfaction with the health workers in general; the stigma is expressed with regard to PLWH child bearing and fear of disapproval. Similar findings have been reported by Harries et al. [52], Nóbrega et al. [21], Orner et al. [20], Mbekenga et al. [49]. This does not only reinforce the CLWH's anxiety and self-judgement that they are currently not adhering to the advice by the health workers, but also led to a critical situation. The informants sought information from other sources and most of them never discussed any issues pertaining to sexual and reproductive health, HIV, and AIDS with their health workers. Comprehensive Model of Information Seeking (CMIS) by Johnson, [53] refers to this as “avoidance behaviour” under the component of information-seeking action. Other studies conducted among PLWH and with both PLWH and health workers by Harries et al. [52], Nóbrega et al. [21], and Orner et al. [20] have found similar avoidance behaviours. Street, [54] and Oosterhoff et al. [55], indicate that avoidance behaviour arising due to cultural differences between a provider and a patient can pose serious challenges to effective communication, especially if one holds negative attitude towards the other's cultural beliefs or if the two differ in their communicative preferences and expectations for the consultation.

Awaring that as the HIV pandemic evolves with PLWH living longer healthier lives they will have continuing sexual and reproductive health desires and needs that must be attended to, the absence of structured personalized communication between CLWH and health workers in the study is a major concern and a missed opportunity for information acquisition. Couples living with HIV can improve the quality of their sexual and reproductive health lives by communicating with health workers [56]. It also calls for the medical fraternity in Malawi to move with the changing times in the field of sexual and reproductive health, HIV, and AIDS. In Vietnam, Oosterhoff et al. [55] indicate that the health workers are apparently more supportive and never question their PLWH's desires to have children because of an understanding of the cultural implications of not having children in the society. A few published papers suggest that when patients fully disclose their concern, expectations, and preferences, health workers are able to assist them more accurately and offer better advice. Furthermore, when the patients receive reproductive information that is culturally adequate and accessible by people with different educational levels, they are more likely to make better informed decisions and feel committed to implementing those decisions [49, 51, 57].

We suggest establishment of personalized communication of reproductive health, HIV, and AIDS oriented topics between health workers and CLWH. Personalized discussions are associated with a good base for decision making in reproductive health [56, 58]. Health workers should offer nonjudgemental dialogue and supportive attitude to clients, which is an ideal situation but seems to be far off because most CLWH indicated that they generally get antipregnancy information from the health workers. Designing information strategies that involve interpersonal, peer-to-peer exchange of information about positive behaviour is likely to be more successful than emphasis on fearful information for example high mortality rates of pregnant women living with HIV and low survival rates of children born to CLWH [5].

Health workers may not be able to give appropriate information due to the guidelines that are used. The guidelines are “silent” or nonprescriptive on sexual and reproductive health information for CLWH who desire to have children [59]. The guidelines may not have been followed probably due to the presence of powerful social norms and stereotyping attitudes regarding childbearing coupled with the health workers' dual loyalty pressures, attitudes, and values. It may also be due to the lack of the guidelines themselves. Therefore, there is a pressing need for the development of prescriptive guidelines for these couples. However, effective prescriptive guidelines can only be developed by, first understanding the CLWH so that the right information is sought from them. This requires not only creativity on the part of the information producers but also attention to what Resnicow et al. [60] refer to as “the surface and deep structure dimensions of cultural sensitivity.” With availability of written procedures on counselling, there is an increased probability that health workers would counsel PLWH on issues related to reproductive health [61].

From the information-seeking trail highlighted, we show that there was a shared decision making process echoing previous findings in the same setting by Mbweza et al. [31] which reflects male involvement in sexual and reproductive health. However, the male informants from both study sites indicated that the men who wanted to be involved in care at the Maternity clinics were often excluded. Similar experiences were identified in low-income suburbs in Tanzania by Mbekenga et al. [62]. The findings are very discouraging especially when Malawi is advocating for male involvement in sexual and reproductive health issues. In some settings, the problem is compounded by lack of guidelines for the health workers on how to handle couples in hospital maternity sections [63]. We argue that upholding stereotypic notions of femininity and masculinity delimits male involvement. Since the health workers are brought up in societies with these patriarchal beliefs, we suggest that the society must be the first to change its beliefs that sexual and reproductive health services clinics are only for women in order to make the men equal partners of the programmes instead of placing the blame solemnly on the health workers.

### 4.5. Methodological Considerations

Case [29] noted that a majority of studies on patient sources of information and information seeking relied on quantitative survey methods. Therefore, a strength in this study is that it provides an example of how a qualitative perspective can capture the context in which information is sourced and also provides a formative foundation for future research. By focusing on the couple in their sociocultural contexts, we found an opportunity to explore how the separate experiences of a husband and a wife are unified to produce a shared and interactive decision: showing that men are involved in sexual and reproductive health, which is a step ahead from the previous studies.

The study lacked information on the actual content of the informant versus health worker interaction because such information was considered private and confidential in nature. This information could have strengthened the results because of its ability to draw conclusions about the more nuanced nature of each informant health worker interaction. Future studies should combine both informants and provider interviews with direct observations of routine consultations after thorough considerations on the ethical issues.

## 5. Conclusion

The advent of ARV's has allowed couples living with HIV to live longer and healthy lives. The reduction in the mother-to-child transmission of HIV encouraged some PLWH to have children. In such a situation, what matters most is that the CLWH make a conscious decision based on the up to date and comprehensive information. Understanding what sources of information CLWH use to make sexual and reproductive decisions can aid policy makers and health workers in providing information that will be accessed and used by CLWH. We argue that if no interventions are put in place, the behaviour might promote recycling of wrong and outdated knowledge among CLWH, which will derail the already achieved ART success stories in Malawi. Empowering already existing and accepted structures, village elders/local counsellors PLWH support group leaders with sexual and reproductive health, HIV, and AIDS knowledge and skills can provide a favourable solution. However, there is a need to harmonize the formal and informal sources of information so that the two complement each other. We argue that no one source of information can be effective to the CLWH; hence, the issue is not to focus on one channel, rather on how, in total, the available sources provide up to date and comprehensive, cultural-specific sexual and reproductive health, HIV, and AIDS information for the couples to base their decisions on.

## Figures and Tables

**Table 1:** Information on marriage and children.

Characteristics	Matrilineal		Patrilineal	
	M	F	M	F
Number of informants in				
1st marriage	1	1	5	3
2nd marriage	8	7	7	4
3rd marriage	0	2	0	1
4th marriage	1	0	0	0

Marital status before current marriage				
Divorced female	7		4	
Divorced male	8		3	
Widow	2		2	
Widower	1		3	

Average number of children informants have ever had				
1st marriage	3		2	
2nd marriage	1		1	
3rd marriage	1		1	
4th marriage	0		N/A	

Couples having children before HIV+ status	9		9	
Couples having children after HIV+ status	9		8	
Couples with HIV+ children	2		0	
